# mTOR signalling pathway in stem cell bioactivities and angiogenesis potential

**DOI:** 10.1111/cpr.13499

**Published:** 2023-05-08

**Authors:** Hamid Lotfimehr, Narges Mardi, Samaneh Narimani, Hamid Tayefi Nasrabadi, Mohammad Karimipour, Emel Sokullu, Reza Rahbarghazi

**Affiliations:** ^1^ Stem Cell Research Center Tabriz University of Medical Sciences Tabriz Iran; ^2^ Department of Applied Cell Sciences, Faculty of Advanced Medical Sciences Tabriz University of Medical Sciences Tabriz Iran; ^3^ Student Research Committee Tabriz University of Medical Sciences Tabriz Iran; ^4^ Koç University Research Center for Translational Medicine (KUTTAM) Istanbul Turkey

## Abstract

The mammalian target of rapamycin (mTOR) is a protein kinase that responds to different stimuli such as stresses, starvation and hypoxic conditions. The modulation of this effector can lead to the alteration of cell dynamic growth, proliferation, basal metabolism and other bioactivities. Considering this fact, the mTOR pathway is believed to regulate the diverse functions in several cell lineages. Due to the pleiotropic effects of the mTOR, we here, hypothesize that this effector can also regulate the bioactivity of stem cells in response to external stimuli pathways under physiological and pathological conditions. As a correlation, we aimed to highlight the close relationship between the mTOR signalling axis and the regenerative potential of stem cells in a different milieu. The relevant publications were included in this study using electronic searches of the PubMed database from inception to February 2023. We noted that the mTOR signalling cascade can affect different stem cell bioactivities, especially angiogenesis under physiological and pathological conditions. Modulation of mTOR signalling pathways is thought of as an effective strategy to modulate the angiogenic properties of stem cells.

## Graphical abstract

Proposed mammalian target of rapamycin (mTOR) activity in the angiogenic potential of stem cells. The modulation of mTOR can affect several downstream factors that regulate different stem cell bioactivities such as differentiation, angiocrine factors, leading to the angiogenic outcome.

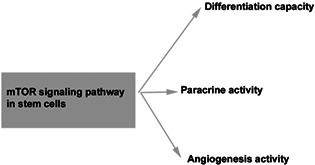

## 1 INTRODUCTION

The existence of several signalling pathways consisting of multi‐state effectors inside each cell can produce specific data in response to various intra‐ and extra‐cellular clues. Following sequential enzymatic reactions and activation of varied scaffold proteins, cells can exhibit functional behaviour.^1^, ^2^ The discovery and advent of stem cells with key biological properties have led to the advancement and progress of therapeutic approaches in human medicine.^3^ Using several mechanisms, stem cells and progenitors can contribute to therapeutic outcomes in injured sites.^4^ The regenerative outcomes are specified by engaging various effectors associated with different signalling cascades.^5^ The tight collaboration between effectors belonging to different signalling cascades activates a specific collection of factors that participate in certain stem cell behaviour.^6^

**FIGURE 1:**
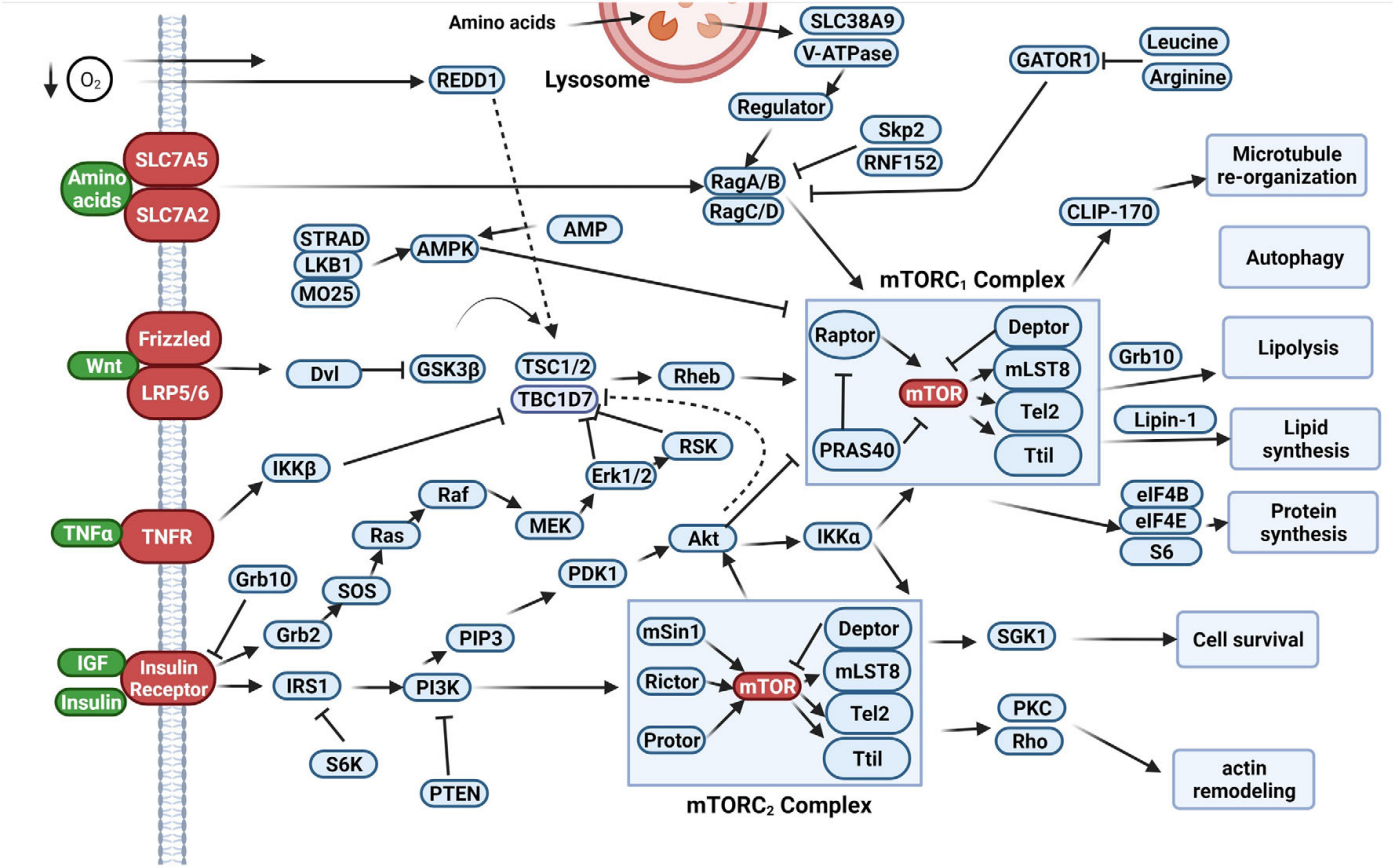
Mammalian target of rapamycin (mTOR) signalling pathway. The mTOR signalling pathway consists of two mTOR complexes, mTORC1 and mTORC2 with the ability to regulate different bioactivities inside the host cells. In upstream, several factors such as amino acids, Wnt, TNF‐α, IGF and insulin can affect the activity of mTOR. The mTORC1 complex regulates microtubule re‐organization, autophagy and lipolysis, lipid and protein synthesis while the mTORC2 complex is involved in cell survival and cytoskeletal re‐modelling.

Among several molecular cascades, the critical role of the mammalian target of rapamycin (mTOR) pathway has been proved in the context of dynamic growths and the regulation of multiple biochemical reactions.^7^ Previous findings are consistent with the fact that the activation of the mTOR signalling axis is associated with the promotion of diverse biochemical reactions which per se are associated with the regulation of transcription, translation, protein synthesis and degradation via the modulation of p70S6K/S6 and 4EBPI/eIF4E.^8^ Metabolic status is a key factor that can affect the activity of the mTOR signalling axis.^9^ The drop of intra‐cellular glucose content to very low levels and the exposure of cells to starvation can provoke the mTOR pathway.^10^ The integration of the mTOR pathway with either intra‐cellular or extra‐cellular signals highlights the importance of this pathway in stem cell regenerative potential under physiological and pathological conditions.^11^ Of note, mTOR is a core‐acting enzyme with a serine/threonine‐protein kinase and belongs to the PI3K family (Figure 1).^8^

**FIGURE 2:**
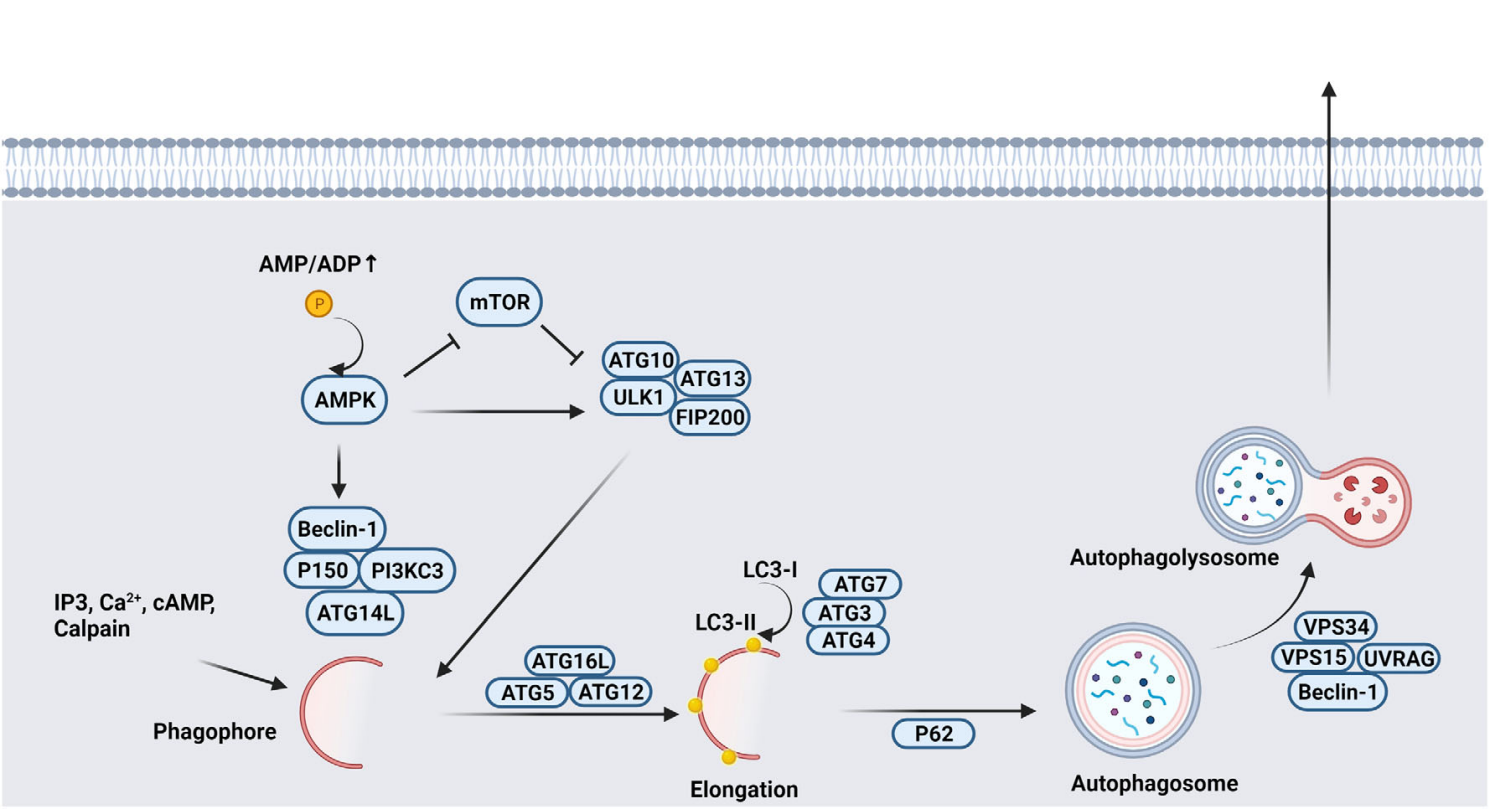
Autophagy machinery following the mammalian target of rapamycin (mTOR) activity. mTOR inhibition can lead to the activation of ATG10, ATG13, ULK1 and FIP200 and formation of primary phagophores. This process is followed by elongation via the participation of the ATG7/ATG3/ATG4 complex and the conversion of LC3‐I to LC3‐II, leading to the formation of autophagosomes. After that, autophagosomes are fused with lysosomes to form autophagolysosomes. In the latter steps, the autophagic cargo is released to the outside of cells or recycled.

It has been shown that mTOR consists of two complexes, namely mTORC1 and two subunits. In terms of molecular structure, mTORC1 is a complex of mTOR, raptor, GβL and deptor. In contrast to mTORC1, mTOR, Rictor, GβL, PRR5, deptor and SIN1 are constituents of the latter complex mTORC2.^10^ From an evolutionary viewpoint, both mTORC1 and 2 are phylogenetically conserved with prominent participation in cell growth and proliferation.^12^, ^13^ To be specific, mTORC1 partakes in the regulation of cell growth and autophagic response while mTORC2 is certainly an important factor in cytoskeletal re‐modelling and actin re‐arrangement (Figures 1 and 2).^14^

Several upstream effectors such as growth factors and insulin, low ATP ratio, hypoxia, Wnt, TNFα and amino acids (glutamine, leucine and arginine) can initiate the mTOR signalling pathway via different transduction pathways.^15^ Mechanistically, different stimulators can activate the mTORC1 signalling pathway. The presence of amino acids, insulin and growth factors can induce the recruitment of mTORC1 to the lysosome surface, and its association with small GTPases namely Rags. Four types of rags, including Rag‐A, Rag‐B, Rag‐C and Rag‐D, participate in the sense of nutrients by mTORC1.^16^ To be specific, Rag GTPases attach physically to the raptor and connects mTORC1 to the lysosome where Rheb, a mTORC1 activator, resides.^16^ Simultaneously, these factors are co‐localized with tuberous sclerosis complex (TSC) as a suppressor of GTPase Rheb activity. The inhibition of the TSC complex and its dissociation coincides with the induction of GTPase activating protein on Rheb, resulting in the phosphorylation of mTORC1 on the lysosomal surface.^17^ The signalling axes associated with mTORC1 fulfil several bioactivities such as suppression of insulin‐related pathways via GRB10, phosphorylation 4EBP1 and ribosomal S6 kinase (S6K) leading to protein synthesis and cell growth.^18^

By phosphorylation of S6K at Thr389, mTORC1 can induce phosphorylation by PDK1 in the kinase domain of S6K. Activated S6K promotes the phosphorylation of ribosomal protein S6 belonging to the ribosomal 40S subunit. It is suggested that mTORC1 can promote the transcription of rRNA via RNA pol I and RNA pol III and enhance ribosome biogenesis via the activation of S6K1.^17^ The over‐expression of T cell differentiation protein 2 (MAL2), belonging to the MAL protein family, induces activation of the MAPK/mTOR signalling pathway, promotes ribosome biogenesis and facilitates proliferation in non‐small cell lung cancer (NSCLC).^19^ The role of mTORC1 in the protein synthesis processes is associated with the phosphorylation of 4E‐BPs. This factor acts as a translation repressor and inhibits translation via the regulation of eIF4E. The phosphorylation of 4E‐BP1 by mTORC1 induces its release from eIF4E and promotes 5′cap‐dependent translation of mRNAs.^17^ S6K phosphorylation by mTORC1 activates as a transcription factor namely sterol regulatory element‐binding proteins (SREBP). It has been indicated that SREBP can induce the expression of key enzymes in the pentose phosphate pathway to accelerate nucleotide synthesis. It was suggested that phosphorylated S6K stimulates pyrimidine synthesis via the promotion of CAD activity.^20^ The activation of SREBP by mTORC1 induces lipogenic gene expression, via activating S6K or inhibits nuclear translocation of LIPIN1 via its phosphorylation.^20^ It has been indicated that mitochondrial elongation factor 2 promotes fatty acid synthesis and cholesterol biosynthesis via activation of ROS/Akt/mTOR signalling pathway and up‐regulation of SREBP.^21^

The close relationship between the mTOR signalling pathway and autophagy has been indicated.^22^ Autophagy is defined as an early‐stage self‐degradation mechanism in response to several insulting conditions. This mechanism can sequestrate misfolded proteins into the intra‐cellular vesicles namely autophagosomes. In the latter phases, the fusion of autophagosomes with lysosomes contributes to the formation of autophagolysosomes and the release of cargo to the cell outside.^23^ Based on the molecular analyses, multiple autophagy‐related proteins (ATGs) are involved in the formation of autophagosomes and the activation of autophagy in a tightly controlled manner.^24^ The modulation of mTORC1 can lead to autophagic recycling of essential components under the situations of cells exposed to starvation and nutrition limitation.^22^ The activation of mTORC1 leads to anabolic reactions inside the cells. Along with these changes, the uptake of various nutrients is enhanced and the autophagic flux is prohibited.^25^ The inhibition of autophagy is orchestrated by phosphorylation of ULK1 at Ser758 and ATG13.^22^ The phosphorylation of ULK1/ATG13/FIP200 complex prohibits the formation of autophagosomes.^22^ Meanwhile, simultaneous phosphorylation of TFEB and MiTF‐TFE family members intensifies these effects.^26^ It should not be forgotten that the cargo recycling by autophagy machinery can activate the mTORC1. These features indicate that autophagy is both upstream and downstream of mTORC1.^22^

In contrast, AMPK promotes autophagy via phosphorylation of ULK1 at different sites (Ser‐555, Ser‐777, Ser‐317 and Ser‐467) and thus triggers autophagic flux.^27^ In cells with energy stress and reduced ATP/AMP ratio, AMPK is stimulated due to allosteric changes which in turn increases the uptake of carbohydrates and lipid β‐oxidation.^28^ The activated ULK1 promotes the phosphorylation of Beclin‐1 in VPS34/Beclin‐1/ATG14 complex. In an alternative way, mTORC1 inhibits autophagy indirectly via the suppression of lysosomal biogenesis, through phosphorylation and inhibition of nuclear translocation TFEB.^29^

Unlike mTORC1, the activation of mTORC2‐related signalling pathways via PKC can orchestrate several functions such as cell division, lineage trans‐differentiation, autophagic and apoptotic responses and post‐translational modification (Figure 1).^18^

It is postulated that in response to growth factors, the activation of mTORC2 contributes to the production of PIP3 via the phosphorylation and activation of AGC kinase family members (PKC, AKT and SGK). In the latter steps, mTORC2 binds to the ribosomes and co‐translationally phosphorylates AKT at Thr450 residue. Alternatively, post‐translational phosphorylation of AKT at Ser473 promotes the lipogenic pathway by activation of SREBP1c.^30^ Furthermore, mTORC2 promotes the production of insulin‐like growth factor 2 (IGF2), by co‐translation and phosphorylation of IGF2 mRNA‐binding protein 1.^31^ mTORC2 can phosphorylate and enhance the activity of YAP and the transcription of YAP‐related genes, leading to the growth and invasion of glioblastoma cells.^32^

Considering the inevitable role of the mTOR signalling pathway in different cell lineages, one could hypothesize that this axis can also affect the dynamic activity of stem cells inside several niches under physiological and pathological conditions, leading to distinctive regenerative outcomes. Here, we aimed to highlight the possible role of the mTOR signalling axis on the dynamic growth of stem cells.

## 2 mTOR PATHWAY ROLE IN DIFFERENTIATION AND STEMNESS MAINTENANCE

As a common belief, stem cells exhibit eminent self‐renewability and differentiation capacity towards different cell lineages.^33^ Because of their quiescent status under physiological conditions, it is thought that stem cells possess low‐rate translation properties despite high cytosolic ribosome levels.^34^ A low‐rate translation makes these cells eligible to maintain themselves in an undifferentiated state.^35^ It has been indicated that high‐rate mRNA expression and translation do necessitate several proteins at the post‐translational level.^35^ Molecular investigations have revealed that either orientation towards certain cell types or maintenance of stemness can be orchestrated via the regulation of ribosome activity and protein synthesis capacity (Table 1).^34^ To be specific, each cell type, especially stem cells, harbour heterogeneous ribosomes with the potential to translate specific kinds of mRNAs in response to protein changes.^36^ Such heterogeneity can lead to distinct interactions between the mRNA‐ribosomal RNA and mRNA‐ribosomal protein which pre‐determines different patterns for translation.^36^ Due to the close association between the mTOR signalling pathway and metabolic activity, mTOR signalling can be tightly involved in the procedure of protein synthesis in stem cells to regulate growth, differentiation and viability.^35^ It was indicated that mTORC1 positively regulates the transcription of ribosomal RNA and proteins and factors associated with ribosome assembly.^37^ The activation of mTOR stimulates global translation rate by engaging effectors 4E‐BP and S6K. Along with these changes, the expression of eIF, eEF and ribosomal proteins are triggered, leading to activation and differentiation of stem cells.^38^ Besides, other possible mechanisms have been proposed for the involvement of the mTOR signalling pathway in the dynamic growth of stem cells. For instance, recent studies have shown that the interaction of Akt with mTOR signalling triggers tendon differentiation of MSCs towards tenocyte‐like cells via the synthesis of type I collagen and ECM deposition.^39^ Akt can activate mTORC1 via the phosphorylation and inhibition of TSC2 and PRAS40.^40^ The inhibition of Akt and mTORC1 impaired the collagen synthesis and tenogenesis of MSCs.^39^ In another experiment conducted by Yao and co‐workers, they showed that the over‐expression of miR‐29a‐3 in human umbilical cord MSCs after activation of PTEN/mTOR/TGF‐β1 signalling led to the secretion of exosomes (Exos) and the expression of tendon‐specific markers.^41^ These features indicated that mTOR signalling can also regulate the paracrine activity of MSCs. It confirmed that miR‐29a‐3 regulates the tenogenesis of MSCs by targeting PTEN and direct interaction of Akt with mTOR.^41^

**TABLE 1:** mTOR signalling pathway and stem cell differentiation and survival.

Cell type	Mechanism of action	References
HSCs	Suppression of mTOR sustains HSCs quiescence and self‐renewal HSCs maintain their stemness via the expression of Sel1L/Hrd1 ERAD complex The inhibition of the Sel1L/Hrd1 ERAD complex leads to the activation of the mTOR signalling pathway and HSC proliferation and stemness removal	111
CD34^+^/CD38^−^ stem cells	Propofol inhibited the differentiation capacity of acute myeloid leukaemia stem cells (CD34^+^/CD38^−^ subsets) In presence of Propofol, the activity of Akt/mTOR, and the Wnt/β‐catenin pathways were diminished.	112
Bone marrow MSCs	The inhibition of the mTOR signalling pathway and ROS production can postpone the premature ageing of bone marrow MSCs Treatment of bone marrow MSCs with IHH factor after the inhibition of mTOR and ROS can delay senescence‐related changes	53
Liver cancer stem cells	CCND1 over‐expression leads to mTOR stimulation and autophagic cell response increasing the number of CD133‐positive cells.	113
Rat bone marrow MSCs	Rapamycin‐treated MSCs with inhibited mTOR and stimulated autophagic response showed increased survival rate and differentiation capacity after being transplantation into the infarcted area.	98
Embryonic stem cells	Active chaperone‐mediated autophagy participates in the balance between stemness features and differentiation capacity by regulating the levels of isocitrate dehydrogenases, α‐ketoglutarate and Histone and DNA demethylases activity.	114
Trophoblast stem cells	Activation of autophagic response and stimulation of Beclin1/Vps34/PIK3R4 complex lead to differentiation capacity The inhibition of lysosomal fusion decreases the expression of Prl3d1, Prl2c2, Prl4a1 and Tpbpα	115
Human umbilical cord‐derived MSCs	MSCs can reduce the apoptosis and autophagy of theca‐interstitial cells in the POI model of a rat via the reduction of ROS and modulation of the AMPK/mTOR signalling pathway	116
Bone marrow MSCs	The promotion of autophagy (mTOR/ULK1) and WNT/β‐catenin pathway increase the osteogenic differentiation of MSCs in the presence of dicalcium silicate nanoparticles	117
Spermatogonial stem cells	The modulation of mTOR activity via AKT and P53 results in autophagy response and resistance of spermatogonial stem cells against Busulfan	118
Osteoblasts and macrophages	Induction of autophagy via rapamycin and modulation of PI3K/AKT/mTOR signalling pathway blunts hydrogen sulphide‐induced osteoclastogenesis and apoptosis of mature osteoblasts The effect of RANKL on osteoclastogenesis is reduced after the induction of autophagy	119
NSCs	Well‐controlled autophagy stimulation can promote NSCs differentiation into mature neurons in the presence of electrical stimulation via the activation of the PI3K/AKT/CREB pathway and suppression of the mTOR signalling pathway	120
Spermatogonial stem cells	The activation of autophagic response and inhibition of PI3K/Akt/mTOR axis increased the spermatogenesis capacity of spermatogonial stem cells in the presence of retinoic acid	121

Abbreviations: CCND1, Cyclin D1; ERAD, endoplasmic reticulum‐associated degradation; HSCs, haematopoietic stem cells; IHH, Indian Hedgehog; MSCs, mesenchymal stem cells; mTOR, mammalian target of rapamycin; NSCs, non‐small cells; PIK3R4, phosphoinositide‐3‐kinase regulatory subunit 4; RANKL, receptor activator of nuclear factor‐kappa‐β ligand; SEL1L, adaptor subunit of ERAD E3 Ubiquitin ligase.

It was indicated that MSCs can limit the differentiation of CD4^+^ T cells towards T helper 17 cells (Th17) via the regulation of the mTOR pathway. The modulation of mTOR signalling is associated with regulatory T cell (Treg) and Th17 differentiation through the activation of STAT3 or STAT5 and HIF‐1α.^42^ PI3K/AKT/mTOR/p70S6K axis plays a role in the differentiation of Th2 and Treg cells, and inhibition of the mTOR pathway, involving in the maintenance of Th2/Treg balance.^43^ Importantly, the suppression of mTORC1 kinase activity reduces the differentiation of intestine Caco‐2 cells into functional enterocytes. Selective activation of upstream effector AMPK can inhibit mTORC1 via phosphorylation of Ser1387 residue.^44^ Therefore, one could hypothesize that mTORC1, in contrast to mTORC2, is not essential to the promotion of cell differentiation towards specific lineages. Whether and how underlying mechanisms are engaged after the promotion of the mTOR signalling pathway is under investigation. Ionic stimulation of the mTOR pathway via magnesium supplementation has been shown to trigger myogenic differentiation of aged muscle progenitor cells.^45^ It is thought that there is a close relationship between the tyrosine kinase activity of the IGF‐1 receptor and the AKT/PKB‐mTOR pathway, leading to the promotion of stem cell differentiation towards myocytes.^46^ It is important to mention that the mTOR pathway is touted as a pleiotropic cascade in the regulation of stem cell differentiation towards several lineages. For example, in a study increased intra‐membrane distribution of GPR30 provoked PI3K/AKT/mTOR and enhanced osteogenesis in periodontal ligament stem cells.^47^ Shikonin, one of the derivatives of naphthoquinone, induces odontoblastic differentiation of dental pulp stem cells through the AKT–mTOR signalling pathway and expression of CD44.^48^ Using anti‐CD44 monoclonal antibody, the levels of phosphorylated‐mTORC1, phosphorylated‐mTORC2 and phosphorylated‐Akt were reduced, leading to enhanced differentiation of HL60, THP‐1 and KG1a leukaemic cells and tumour growth suppression.^49^ These data reinforce the idea that the regulation of CD44 and inhibition of PI3K/Akt/mTOR pathway are strategic plans to control the propagation of leukaemic cells.

Under starvation conditions, accumulated pyruvate with an increased AMP/ATP ratio expedites the mesodermal differentiation of human ESCs via the stimulation of AMPK.^50^ Data indicated that elevated pyruvate can blunt glycolytic capacity and glycolysis. In line with these changes, the components of tricarboxylic acid were increased, indicating stimulated mitochondrial oxidative phosphorylation.^50^

Upon myogenic differentiation, syndecan‐3 reduces the activity of insulin receptor after interaction with AKT/mTOR pathway.^51^ It was suggested that modulation of miR‐143‐3p and miR‐100‐5p via the mTOR pathway is involved in driving human MSC fate for tissue regeneration.^52^ Both miRNAs act as mechanosensitive factors and their expression can be changed in response to matrix stiffness and RhoA activity.^52^ It was shown that co‐transfection of encapsulated MSCs with co‐transfection of miR‐100‐5p and miR‐143‐3p inside 3D gelatin–polyethylene glycol hydrogel diminished protein levels of mTOR, Rictor and Larp1 and exerted rapamycin‐like effects.^52^ The inhibition of mTOR and its effectors increases the osteogenic potential of MSCs with the deposition of hydroxyapatite and alkaline phosphatase activity.^52^ In an experiment, it was indicated that an Indian Hedgehog (IHH) shortage leads to skewed differentiation in bone marrow‐derived mesenchymal stem cells (BMSCs), which can be modified by inhibition of mTOR and ROS.^53^ The incubation of mouse MSCs with IHH siRNA led to premature ageing (β‐galactosidase↑) and skewed differentiation.^53^ The activation of mTOR and accumulation of ROS is associated with senile changes and skewed differentiation in MSCs.^53^ Concerning several bioactivities, recent investigations have confirmed the crucial role of the mTOR signalling pathway in the orientation and phenotype acquisition of several stem cell types.^7^

CSCs are a small fraction of cells within tumour parenchyma with prominent tumourigenesis and metastatic activity.^54^ These cells exhibit unique self‐renewal and stemness features via the expression of certain factors such as CD44^+^/CD24^−^ and CD133^+^, resulting in resistance to chemotherapeutic agents.^54^ Using tissue microarray, it was suggested that mTOR is highly expressed in 74% of patients with NSCLC.^55^ Likewise, inevitable roles of PI3K/Akt/mTOR and IL‐8 were indicated in CSCs isolated from hepatic tissue.^56^ Based on the data, rapamycin can diminish the positivity of liver CSCs in terms of CD133 and EpCAM and spheroid formation.^56^ Treatment of pancreatic tissue CD133^+^ CSCs with rapamycin reduces survival rate and stemness features with simultaneous loss of CD133.^57^ The down‐regulation of PI3K/AKT/mTOR with the loss of clonogenic capacity was achieved in breast CSCs after exposure to flavonoid quercetin.^58^ These studies highlight the importance of the mTOR signalling pathway in the phenotype acquisition of CSCs within the tumours in which the loss of mTOR is integral to the loss of stemness feature. It was suggested that prolonged TGF‐β treatment activates mTOR and epithelial‐mesenchymal transition (EMT), resulting in stemness maintenance and tumour expansion. By applying a bitopic mTOR blocker, the survival rate and stemness feature were reduced via the inhibition of TGF‐β.^59^ In an experiment conducted by Katsuno and colleagues, deprivation of HMLE cells from TGF‐β blunted EMT rate with the reduction of CD44^+^/CD24^−^ cells, and downregulation of certain genes such as *NANOG*, *POU5F1* and *SOX2*.^59^ Data have pointed to the fact that the inhibition of mTOR in CSCs is an effective way to control tumour propagation via the reduction of CSCs and stemness removal. It should not be neglected that mTOR signalling pathway has synergism with other molecular pathways to control dynamic activity of CSCs.^60^ Friedman and co‐workers indicated that treatment of glioblastoma U87 and LN18 cells with rapamycin and all‐trans retinoic acid increases differentiation of CSCs and loss of nestin. These features coincided with the reduction of neurosphere diameter and migration properties.^60^ In line with this statement, the inhibition of mTOR using rapamycin improved the suppressor activity of Fbxw7 gene.^61^ The activity of Fbxw7 is associated with the ubiquitination of different oncoproteins in which homozygous or heterozygous ablation of this gene can contribute to several malignancies.^61^ Liu et al. claimed that temporal inhibition of mTOR delayed tumour development and blunted the loss of Fbxw7 factor in *Fbxw7* ^*+/−*^ mice.^61^ Simultaneous inhibition of mTOR and treatment of human breast cancer specimens with tamoxifen led to stemness reduction, promotion of mesenchymal to epithelial transition (MET), and sensitivity to chemotherapeutic agents.^62^ Some data revealed that inhibition of multiple mTOR signalling pathway effectors is another strategy for achieving regenerative outcomes. For instance, dual inhibition of mTOR and PI3K contribute to the reduction of colorectal SW620 CSC population and MET rate.^63^ mTORC1/S6K1 pathway is also involved in the regulation of CSC stemness.^64^, ^65^ The activation of mTORC1/S6K1 axis promotes the phosphorylation of Gli1 belonging to the Hedgehog pathway and increases the release of Gil1 from an endogenous inhibitor SUFU. The mTORC1/S6K1 axis can also reduce the degradation of Gil1 via GSK3β. Nuclear translocation of GSK3β is tightly controlled after the activation of mTORC1/S6K1 pathway, leading to the stabilization of stemness factor c‐Myc.^64^, ^65^

Like mTORC1, the regulatory role of mTOC2 has been documented in terms of CSC stemness. It was suggested that mTORC2 can inhibit FoxO upon phosphorylation of Akt. Following Akt phosphorylation, nuclear translocation of FoxO is decreased, resulting in the release of c‐Myc. Along with these changes, GSK3β is stabilized in the cytosol to activate β‐catenin and Gli2 and up‐regulate the expression of *SOX2*, *OCT4* and *Nanog* in CSCs.^64^, ^65^, ^66^ Hypoxic conditions within the tumour niche preserve the stemness features of CSCs. The elevation of HIF‐2α in hypoxic tumour cells increases the number of CD44^+^/CD24^−^ MDA‐MB‐231 cells via the modulation of the PI3K/AKT/mTOR axis.^67^ Taken together, these data confirm the key role of mTOR and relevant effectors in the maintenance of stemness and CSC population within the tumour niche. Of course, due to the complexity and existence of various effectors, it is postulated that the mTOR pathway can directly or in collaboration with other molecular pathways affect the dynamic growth and differentiation capacity of CSCs.

## 3 ROLE OF mTOR SIGNALLING ON ANGIOGENESIS CAPACITY

The term angiogenesis or neovascularization refers to the formation of nascent blood vessels from the pre‐existing network.^68^ It is thought that angiogenesis is a fundamental procedure with the participation of multiple cells. In response to the secretion of several pro‐angiogenesis factors, endothelial cells (ECs) proliferate and generate nascent blood vessels under physiological and pathological conditions.^69^ In a complementary mechanism known as vasculogenesis, the differentiation of bone marrow endothelial progenitor cells, namely EPCs, into mature ECs and the formation of primitive vascular plexus can support blood into certain sites.^70^ Irrespective of vessel formation via neo‐angiogenesis or vasculogenesis, the activation of certain signalling pathways and balances between pro‐ and anti‐angiogenic factors can pre‐determine the fate of vascular generation inside the body.

Previous findings are consistent with the fact that persistent activation of mTOR promotes angiogenesis. In support of this notion, the inhibition of mTOR1 and 2 via the specific blocker can prohibit angiogenesis in mouse models of retinoblastoma xenograft.^71^ In vitro data have indicated that TAK‐228, an FDA‐approved mTOR compound, can reduce the tubulogenesis activity of human retinal ECs in a dose‐dependent manner. Further analyses have revealed that TAK‐228 exerted a dual inhibition of retinoblastoma cell growth and angiogenesis properties. Upon the incubation of retinoblastoma RB355 cells and retinal ECs with TAK‐228, phosphorylation of mTOR was reduced at Ser2448. Along with these changes, phosphorylation of S6K1 and 4EPB1, and AKT was blunted in both cell types.^71^

The reciprocal interaction between EPCs, ECs and haematopoietic progenitor cells (HPCs) is critical in EC activity. Recent data indicated that ageing‐related changes can alter crosstalk between endothelial niche and haematopoietic cells via the inhibition of the mTOR signalling pathway.^72^ Ramalingam and co‐workers indicated that conditional deletion of mTOR in young mice ECs promotes the ageing of HPCs. Along with these changes, the levels of VEGF were reduced and the RTK signalling pathway was inhibited. Interestingly, the transplantation of wild‐type HPCs to *mTOR* ^−^/^−^ mice restored bone marrow activity.^72^ The inhibition of mTOR signalling is associated with a significant reduction of endothelial CD31 marker which can per se affect both angiogenesis and osteogenesis.^73^ Using mTORC1/2 inhibitors, WYE‐132 and AZD8055 compounds, Cong et al. indicated that the number of CD31^+^ and α‐SMA^+^ vessels was decreased in a mouse xenograft model that received lung cancer H1975 cells and U87MG glioblastoma cells.^73^ These data coincided with the reduction in the phosphorylated form of Akt, Erk and S6 factors.

It was suggested that cytoskeletal re‐modelling and morphological adaptation are critical issues involved in the angiogenesis capacity and the formation of aligned EC layers.^74^ In this regard, the collaboration and cross‐talk between VEGF, Notch and mTOR signalling pathways are important.^74^ Of note, co‐inhibition of PI3K‐Akt and mTORC1 in the presence of angiogenic factors such as VEGF can promote endothelial lineage adaptation with significant elongation.^74^ To be specific, the inhibition of these pathways in ECs can induce elongated forms of ECs even in the presence of low levels of VEGF. These data support the notion that PI3K‐Akt and mTORC1 have negative effects on EC elongation and induce specific morphology. Of course, it should not be forgotten that the inhibition of mTOR can affect other adaptive molecular signalling pathways leading to hypermetabolic activity in certain stem cell lineage like haematopoietic stem cells (HSCs).^75^ In mTOR knockout mice, the stimulation of ERK‐MNK‐eIF4E‐RNA polymerase II‐c‐Myc can compensate for mTOR‐related activity in HSC proliferation capacity by influencing global gene expression.^75^ Fan and co‐workers indicated that mTOR deficiency can inhibit the colony‐forming properties of HSCs. In line with these changes, the number of HSC subsets at G0 was significantly reduced while active cells at phases of G1 and S/G2/M increased, indicating the uncontrolled expansion of HSCs.^75^ RNA sequencing showed that the expression of genes associated with proliferation such as *c‐Myc*, *Pim1*, *Fos*, *Dusp1* and *Jun* was stimulated in mTOR‐deficient HSCs.

Recent findings extended our understanding of mTOR's impact on angiogenesis potential via the regulation of cell metabolism and biochemical reactions.^76^ The inhibition of AKT and mTOR expression can negatively control the activity of enzymes related to the glycolytic pathway and angiogenesis in tumour cells.^76^ For example, in bladder cancer cells the inhibition of Hepatitis B X‐interacting protein (HBXIP), involved in centrosome duplication, suppressed the angiogenesis capacity via the inhibition of VEGF and erythropoietin. Liu and colleagues indicated that phosphorylation and expression of mTOR and AKT were reduced in HBXIP‐inhibited tumour cells. Besides, the activity of glycolytic enzyme proteins was diminished, leading to a reduction of glucose consumption and lactate content.^76^ In tumour ECs, both aerobic oxidation and most probably glycolytic pathways are activated. Therefore, it is logical to hypothesize that the inhibition of glycolysis, the main source of energy production mechanism in cancer cells, can lead to the suppression of angiogenesis and thus EC atresia.^77^ It is well‐recognized that the activity of certain glycolysis enzymes such as enolase‐1 (α‐enolase) can affect angiogenesis. Shu et al. claimed that the inhibition of α‐enolase is associated with the loss of stemness properties, migration and dynamic growth in pulmonary CSCs.^77^ This enzyme can stimulate the stemness features in CSCs via the phosphorylation of 4EBP1, S6K and mTOR. Commensurate with these data, α‐enolase is proposed as a valuable target molecule in cancer therapy. Whether and how mTOR modulation can preserve stemness features or induce mature phenotype in certain lineages needs further investigation.^78^ Besides the fundamental role of glycolysis in angiogenesis, the reduction of mitochondrial respiration after the accumulation of harmful free radicals and insidious oxidants occurs. Under such circumstances, the activity of Akt/mTOR is prominently diminished via the phosphorylation of AMPK.^79^ In a similar work, the incubation of amniotic‐derived epithelial cells Exos with fibroblasts or ECs induced angiogenesis potential via PI3K/AKT/mTOR axis under diabetic conditions, indicating the integration of PI3K pathway with mTOR signalling cascade.^80^ Based on recent data, it seems that an array of signalling biomolecules can affect the activity of the PI3K/AKT/mTOR axis. For instance, the expression of type I and II polycystin proteins following in polycystic renal tissue correlates with hyper‐phosphorylation of Akt, activation of the p110γ subunit of PI3K and VEGF.^81^ Like mature ECs, the importance of the PI3K/AKT/mTOR axis has been recently clarified in the vasculogenic capacity of EPCs after exposure to Celastrol belongs to quinone methides.^82^ The reduction of phosphorylated PI3K/AKT/mTOR can blunt vascular mimicry in Celastrol‐treated EPCs coinciding with inhibition of the VEGFR‐2/Angiopoietin‐2/VEGF axis, indicating the critical role of this molecular axis in the function of progenitor and adult endothelial lineage.^82^

**FIGURE 3:**
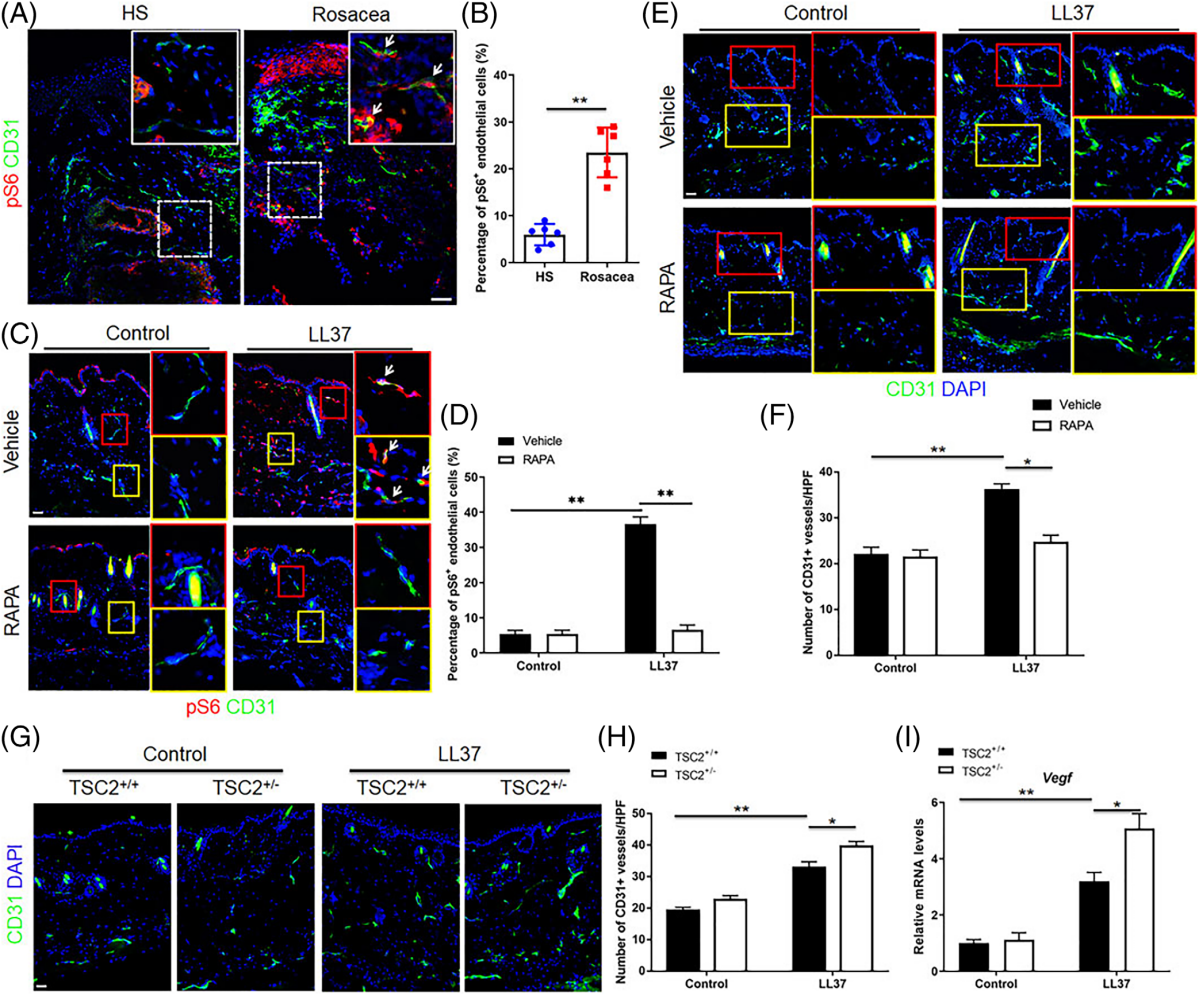
The role of mammalian target of rapamycin (mTOR) in cutaneous tissue angiogenesis (A–H). In vivo angiogenesis was induced using an active form of Cathelicidin namely LL37. Immunofluorescence staining of CD31 and pS6 was performed in rosacea patients and compared to the normal samples (HS) (A, B). Immunostaining of CD31 and pS6 in mice cutaneous samples after treatment with LL37 and rapamycin (C, D; White arrows: CD31^+^ cells). Semi‐quantitative measuring of CD31 vessels in LL37‐treated rosacea‐like mice after treatment with rapamycin (E, F). Semi‐quantitative measuring of CD31^+^ vessels in control and TSC2^+/−^ knockdown mice after treatment with LL37 (G, H). Real‐time PCR analysis of VEGF in normal and TSC2^+/−^ knockdown mice treated with LL37. Scale bar: 50 μm. Student's *t*‐test (B). One‐way ANOVA with Bonferroni's post‐hoc test (D, F, H and I). *p < 0.05; **p < 0.01^84^ (Copyright 2021, Frontiers in Cell and Developmental Biology).

Stem cell‐derived Exos harbour several signalling molecules which are important in the regeneration of damaged tissues. Of these factors, miRNAs are short‐length genetic materials that can be transferred via Exos and alter the activity of certain effectors inside the acceptor cells. It has been documented that injection of BMSC Exos containing miR‐29c into a mouse model of experimental infarction led to an improved regeneration of the ischemic area via the stimulation of autophagy and activation of the PTEN/Akt/mTOR axis.^83^ Peng and co‐workers indicated the elevation of pS6, a mTORC1 activator, along with increased CD31^+^ ECs in the cutaneous tissue of rosacea‐like mouse models and human counterparts.^84^ The inhibition of mTOR using rapamycin resulted in the reduction of excessive CD31^+^ vascular beds. Interestingly, the number of CD31^+^ ECs and VEGF levels were high in the skin of TSC2^+/−^ mice when compared to the TSC2^+/+^ control group (Figure 3). These features indicated that the loss of inhibition on mTOR can lead to aberrant angiogenesis under pathological conditions. In an experiment conducted by Ding and co‐workers, they explored the role of Deptor, a mTORC1 suppressor, which was investigated on angiogenesis potential.^85^ It has been indicated that the levels of VEGF, HIF‐1α and CD31 were increased in cardiac, hepatic and renal tissues in Deptor^−^/^−^ mice.^85^ Despite the existence of scientific documents regarding the stimulatory effects of mTORC1 on angiogenesis, in some circumstances like cancer niche, the sole inhibition of mTORC1 cannot lead to sufficient blockade of blood supplementation.^86^ In this regard, the application of KU0063794 with dual inhibition of mTORC1/mTORC2 can be an efficient strategy to inhibit the angiogenesis potential of ECs.^86^ In ECs exposed to KU0063794, EC elongation and tubulogenesis capacity were significantly diminished. These features coincided with abnormal accumulation of F‐actin filaments. Likewise, KU0063794 has the potential to abort the angiogenic potential of murine ESCs via simultaneous inhibition of mTORC1/mTORC2.^87^ Similarly, the inhibition of mTORC1/mTORC2 using ZJQ‐24, an indole hydrazide compound, led to the inhibition of eIF4F, dephosphorylation of AKT and vascularization in tumour parenchyma.^88^ Previous data have indicated the collaboration of the mTOR signalling pathway with other molecular cascades is important in terms of angiogenesis. For example, the attachment of ligand CXCL12 to cognate receptor CXCR4 promotes CXCR4/PI3K/mTORC2/Akt and stimulates the angiogenesis and migration of ECs via the mTORC1 axis.^89^

Under inflammatory conditions, several cytokines such as prostaglandin E2 exert pro‐angiogenic effects via the activation of mTORC2. This prostaglandin stimulates the pro‐angiogenic factors such as VEGF, FGF and so forth, and certain adhesion molecules like PECAM‐1 in tumour ECs.^90^ There are controversial data related to the exact role of mTORC1 and/or mTORC2 on angiogenesis. For example, it was suggested that the suppression of mTORC2, but not mTORC1, can blunt the angiogenesis properties in ECs.^91^ A large number of pro‐angiogenic factors and receptors such as VEGF, IGF‐1, FGF, VEGFR‐2 and insulin can orchestrate angiogenesis capacity in ECs via engaging mTORC2 independent of mTORC1 activation. From molecular aspects, the attachment of growth factors to relevant RTKs promotes the phosphorylation of CD146 and leads to the interaction of CD146 KKGK motif with the Rictor of mTORC2. This physical interaction stabilizes mTORC2 and stimulates angiogenesis in PI3K/mTORC1 independent manner.^92^

**FIGURE 4:**
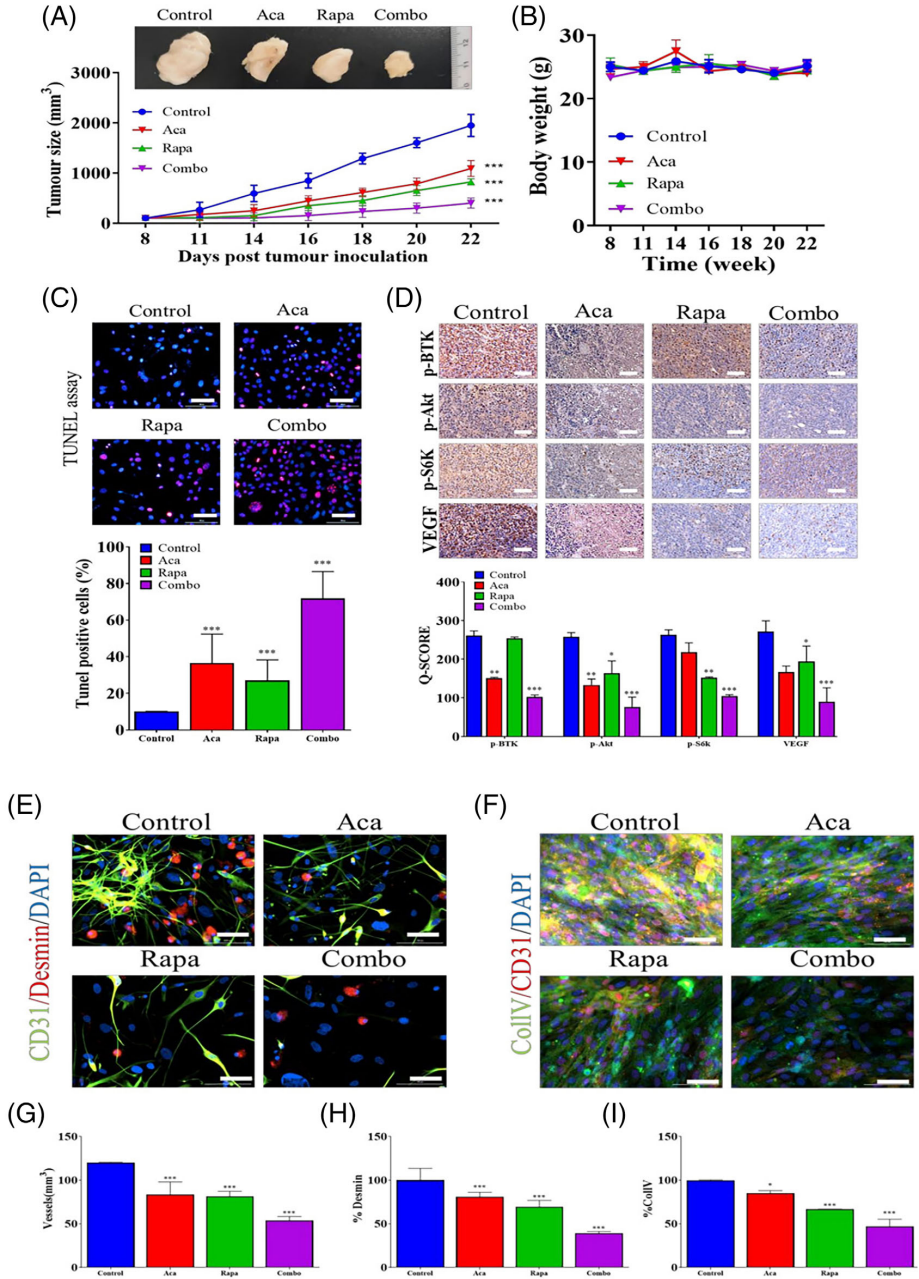
Pro‐angiogenesis properties of rat bone marrow mesenchymal stem cells (MSCs) in cardiac ischemia/reperfusion model. GFP‐expressing MSCs were pre‐treated with rapamycin and transplanted into the affected sites in cardiac tissue. Immunofluorescence imaging revealed that GFP^+^ MSCs can express CD31 (white arrows) and align in the vascular lumen, indicating the stimulatory role of mammalian target of rapamycin inhibition on endothelial lineage differentiation of MSCs. Scale bar = 100 μm. *p < 0.01 related to control; ^#^ *p* < 0.01 related to MSC group (*n* = 6)^94^ (Copyright 2021, Pharmaceuticals).

The molecular identities of stem cells in different conditions are associated with the crosstalk of the mTOR signalling pathway with other molecular cascades.^93^ For instance, the neural differentiation capacity of small peptide ghrelin was indicated on adipose‐derived MSCs orchestrated via AKT/mTOR and β‐catenin pathway. The activation of the AKT/mTOR axis can stimulate nuclear β‐catenin and phosphorylated GSK‐3β.^93^ Of note, the suppression of the AKT/mTOR axis or β‐catenin can deteriorate the neurogenesis capacity of ghrelin on MSCs. These data show that the collaboration of the mTOR signalling pathway is vital to orient the stem cells towards certain lineages. In another study, the application of EC Exos loaded with miR‐29a‐3p‐specific agonist promoted the healing procedure in Achilles tendon injury in a rat model. Molecular investigation revealed the participation of TGF‐β1 and mTOR signalling pathways as well as PTEN effectors in tendon regeneration via paracrine activity.^41^ In glioma CSCs, the close collaboration between BTK and mTOR/VEGF axis can reduce stemness potential and angiogenesis capacity via the regulation of CD31^+^ cells, indicating the critical role of BTK/mTOR/VEGF in the progression of tumour mass via vasculogenesis (Figure 4).^94^

**FIGURE 5:**
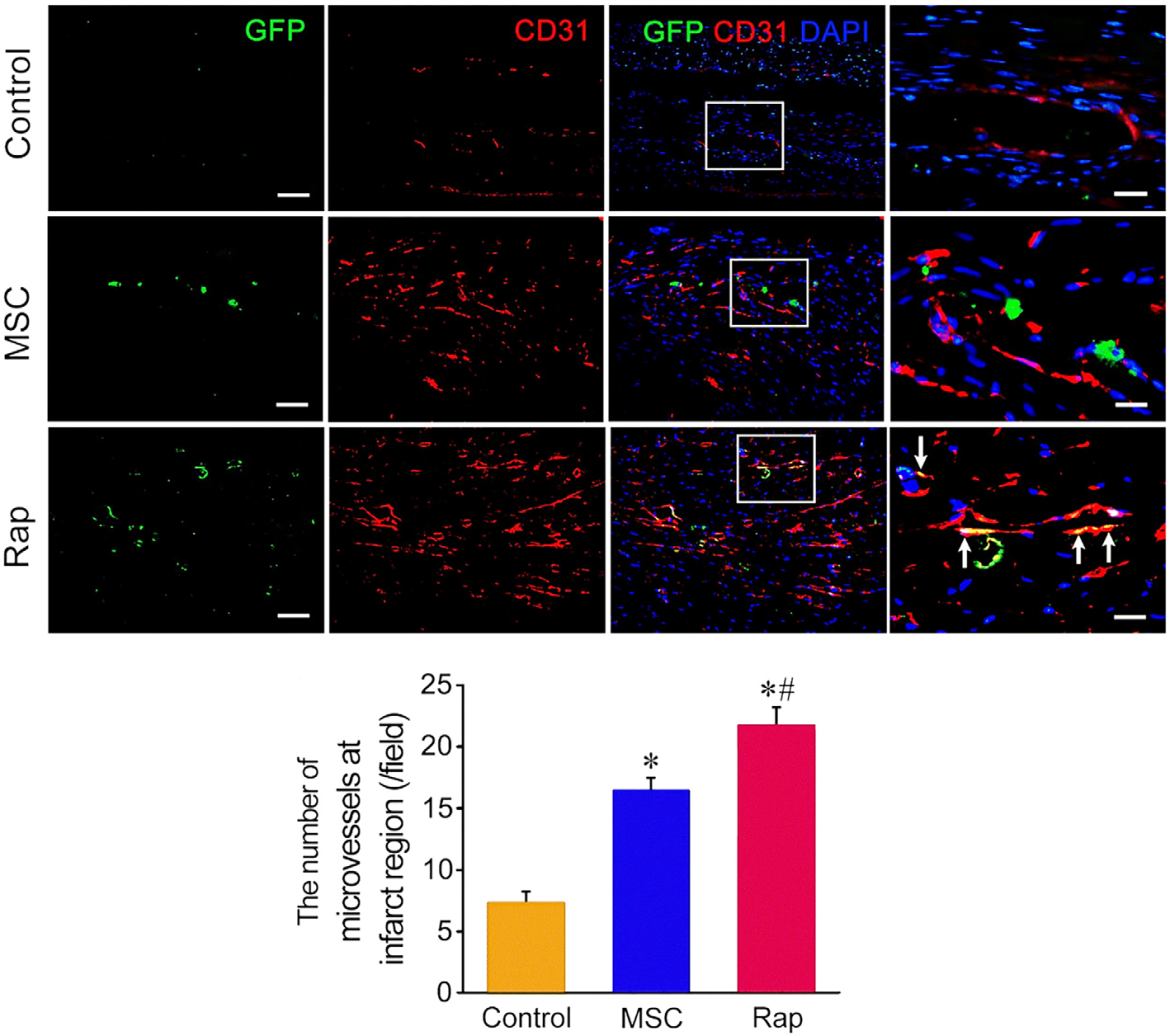
Pro‐angiogenesis properties of rat bone marrow mesenchymal stem cells (MSCs) in cardiac ischemia/reperfusion model. GFP‐expressing MSCs were pre‐treated with rapamycin and transplanted into the affected sites in cardiac tissue. Immunofluorescence imaging revealed that GFP^+^ MSCs can express CD31 (white arrows) and align in the vascular lumen, indicating the stimulatory role of mTOR inhibition on endothelial lineage differentiation of MSCs. Scale bar = 100 μm. *p < 0.01 related to control; ^#^ *p* < 0.01 related to MSC group (*n* = 6)^98^ (Copyright 2020, Stem Cell Reviews and Reports).

The modulation of autophagy is another mechanism by which mTOR can affect the angiogenesis potential in stem cells.^70^ More in‐depth investigations regarding the role of autophagy on the angiogenesis capacity of stem cells have led to controversies in the field.^95^ Incubation of ECs with MSC‐derived Exos promotes angiogenesis capacity via the promotion of tubulogenesis and EC migration.^96^ In a study conducted by Xia and colleagues, the injection of MSC Exos promoted ischemic stroke injury via the inhibition of autophagy response following the activation of STAT3.^96^ It was shown that the inhibition of Apelin, an endogenous ligand with a G protein‐coupled receptor; accelerates MSC senescence indicated by the over‐activity of beta‐galactosidase activity.^97^ It was suggested that the stimulation of Apelin rejuvenated senile MSCs and promoted angiogenic capacity via phosphorylation of AMPK, inhibition of mTOR, and induction of autophagy.^97^ Along with these changes, an intra‐cellular increase of ATG7 and 12 coincided with an increased LC3 II/I ratio occurs to exclude exhaust materials from the host cell.^95^ It is believed that the stimulation of autophagy in MSCs can increase their resistance against short‐ and long‐term hypoxic conditions after transplantation into the target sites. In line with these statements, pre‐treatment of MSCs with autophagy stimulators such as rapamycin can increase cell retention number, activate paracrine mechanisms and promote angiogenesis capacity via the secretion of VEGF, HGF, IGF, SCF and SDF‐1α (Figure 5).^98^ Likewise, Jeong and co‐workers indicated that the promotion of autophagy in ECs can increase blood supply and vessel growth into the ischemic area in the mouse hind limb.^99^

Like autophagy, the integrity of the mTOR complex and lipolysis has been previously indicated.^100^ To be specific, the activity of the mTOR complex, especially mTORC1, restrains the lipolysis rate in adipocytes and prohibits systemic hypercholesteremia and hyperlipidemia.^100^ MSCs transplantation can diminish post‐hepatectomy liver failure via the regulation of fatty acid β‐oxidation and intra‐hepatocyte lipid content. These effects were tailored after the activation of the mTOR signalling pathway. Besides, local production of interleukin‐10 via injected MSCs is a compensatory mechanism to regulate immune cell function and promote angiogenesis in the injured areas.^101^

## 4 mTOR AND PARACRINE ANGIOGENESIS ACTIVITY OF STEM CELLS

**FIGURE 6:**
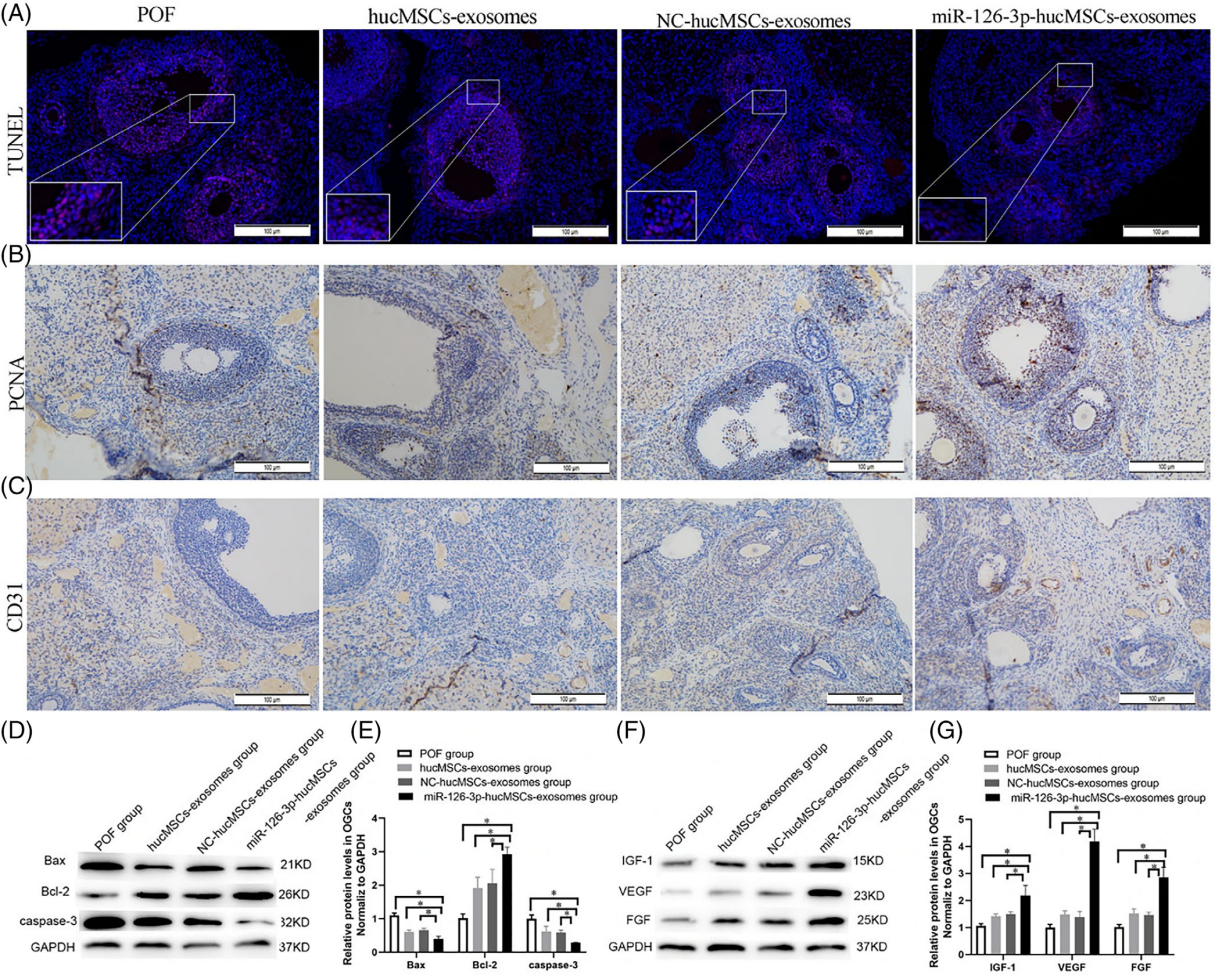
Evaluation of angiogenesis and apoptosis rate in ovarian tissue of rats with premature ovarian failure (POF) (A–G). Immunofluorescence imaging revealed that the number of TUNEL^+^ cells was reduced in the POF rats that received miR‐126‐3p‐bearing Exo (A). Immunohistochemistry staining indicated increased PCNA cell number in ovarian follicles, indicating the number of proliferating cells (B). In line with these changes, the number of CD31^+^ cells was increased in the POF rats that received 126‐3p‐bearing Exo (C). Western blotting indicated the suppression of apoptosis‐related Bax, Caspase 3, and induction of anti‐apoptotic factor Bcl‐2 (D, E). 126‐3p‐bearing Exo increased protein levels of FGF, VEGF and IGF‐1 in the ovarian tissue (*n* = 10). One‐way ANOVA analysis. hucMSC, human umbilical cord MSCs derived exosomes; NC, negative control; PCNA, Proliferating cell nuclear antigen. *p < 0.05^110^ (Copyright 2022, Stem Cell Research & Therapy).

In addition to the differentiation capacity of stem cells towards ECs, the production and release of several angiogenic factors can promote the formation of de novo blood vessels in the target sites.^102^ Emerging data have confirmed the close association between mTOR signalling and the release of factors via nano‐sized vesicles namely Exos.^103^ Exos with endosomal origin range between 30 and 100 nm and are produced via the invagination of the vesicular membrane into the lumen of MVBs. Molecular investigations revealed that Exos contains certain cargoes with several types of growth factors that are involved in inter‐cellular communication.^104^ Due to the complexity of the mTOR signalling cascade and Exos biogenesis, data have confirmed the interplay between these two pathways via shared signalling effectors.^105^, ^106^ To be specific, continuous activation of mTOR1 can lead to intra‐cellular accumulation of CD63^+^ vesicles and inhibition of Exos release from origin cells in in vitro and in vivo conditions. By contrast, the suppression of mTOR1 activity via rapamycin or other growth factors can yield the opposite outcome.^106^ In an experiment, the inhibition of mTOR1 with a specific inhibitor, Torin‐1, increased MVBs interaction with lysosomal marker LAMP1 and Exo release. In line with these changes, the co‐localization of mTOR1 with VPS16 and LAMP1 is inhibited, suggesting a critical role of ceramide‐mTOR signalling in Exo secretion.^103^ In addition to the modulatory effect of mTOR on the Exo abscission rate, emerging data have proved that Exos can also regulate the function of the mTOR axis after entry into the target cells.^107^ It has been indicated that Exos isolated from miR‐26a‐expressing MSCs promoted axonal regeneration in a rat model of spinal cord injury via the modulation of the PTEN/AKT/mTOR axis.^107^ This strategy can be used by cancer cells to stimulate angiogenesis into the tumour parenchyma. In an experiment, it was suggested that hepatocellular carcinoma development and expansion are mediated by angiogenesis via the activation of the PI3K/Akt/mTOR pathway in HUVECs. To this end, cancer cells can release Exos enriched in SNHG16 which can increase the expression of resistant factor GALNT1.^108^ Of note, the close interaction of the mTOR signalling pathway with angiogenesis effectors can facilitate the modulatory effects of Exos. For instance, miR‐100‐loaded Exos can efficiently regulate the mTOR/HIF‐1α/VEGF pathway and blunt angiogenesis in breast cancer cells.^109^ In an experiment conducted by Qu and colleagues, they examined the angiogenic effect of human cord blood MSC Exos on a rat model of premature ovarian failure (Figure 6).^110^ Exos isolated from miR‐126‐3p over‐expressing MSCs promoted angiogenesis via the acceleration of the PI3K/Akt/mTOR signalling axis. These Exos induced the phosphorylation and synthesis of mTOR, Akt and PI3K in granulosa cells. Along with these changes, the administration of miR‐126‐3p‐loaded Exos can increase the levels of CD31 vessels and PCNA^+^ cells in the ovarian tissue matrix coinciding with the reduction of TUNEL‐positive cells.^110^ These data show the close relationship between the mTOR signalling pathway and Exo abscission. Due to the existence of numerous signalling biomolecules and factors inside Exos, it is mighty that Exos can easily modulate the effectors related to the mTOR pathway.

## 5 CONCLUSION

It is thought that the mTOR signalling pathway can affect the bioactivity of stem cells, such as dynamic growth, differentiation capacity and angiogenesis potential under physiological and pathological conditions. Due to the complex entity of the mTOR signalling pathway and reciprocal cross‐talk with other signalling cascades, it is difficult to interpret the exact role of this pathway in stem cells in in vitro and in vivo conditions. It would be better for further studies to focus on the determination of angiogenesis capacity and address the exact role of mTORC1 and mTORC2 complexes in the angiogenic potential of progenitors and stem cells. Whether and how these subunits and downstream effectors collaborate and/or blunt their effects remain unknown. Besides, the exact role of the mTOR signalling pathway in normal stem cells and CSCs should be addressed in terms of angiogenesis.

## AUTHOR CONTRIBUTIONS

Hamid Lotfimehr, Narges Mardi, Samaneh Narimani, Hamid Tayefi Nasrabadi, Mohammad Karimipour and Emel Sokullu collected data and prepared the manuscript. Reza Rahbarghazi supervised the study.

## FUNDING INFORMATION

This study was supported by grants from NIMAD National Institute for Medical Research Development (NIMAD; Grant No: IR.NIMAD.REC.1397.512) and Tabriz University of Medical Sciences (IR.TBZMED.VCR.REC.1397.395).

## CONFLICT OF INTEREST STATEMENT

The authors declare that they have no competing interests.
